# The impact of physical distancing on body-image and social media use

**DOI:** 10.1192/j.eurpsy.2021.93

**Published:** 2021-08-13

**Authors:** O. Corazza

**Affiliations:** Department Of Clinical And Pharmaceutical Sciences, University of Hertfordshire, Hatfield, United Kingdom

**Keywords:** Problematic use of the internet, self-distancing, Covid-19, self-image

## Abstract

The current coronavirus pandemic (Covid-19) is posing new critical challenges on mental health due to widespread social alarm as well as long lasting “physical distancing” as a result of public health protection measures or voluntary conduct. In a period of uncertainty, certain rewarding behaviors, such as the use of the Internet, exercise among other coping strategies might have increased considerably. We will share the results of an international cross-sectional investigation on the impact of physical-distancing on such potentially addictive behaviours to mitigate the pandemic effects, while identifying the most risky patterns and vulnerable populations. The studied sample consists of 3161 participants from Italy (41%), Spain (16%), the UK (12%), Lithuania (12%), Portugal (11%), Japan (6%), and Hungary (4%). Results are currently being analysied.

**Disclosure:**

No significant relationships.

